# Comparative Mitogenome Analysis of Two Native Apple Snail Species (Ampullariidae, *Pomacea*) from Peruvian Amazon

**DOI:** 10.3390/genes14091769

**Published:** 2023-09-07

**Authors:** Alejandro Mendivil, Rina Ramírez, Jaime Morin, Jorge L. Ramirez, Raquel Siccha-Ramirez, Ricardo Britzke, Fátima Rivera, Andre Ampuero, Nilda Oliveros, Carlos Congrains

**Affiliations:** 1 Facultad de Ciencias Biológicas, Universidad Nacional Mayor de San Marcos, Av. Carlos Germán Amezaga 375, Lima 15081, Peru; alejandro.mendivil@unmsm.edu.pe (A.M.); jramirezma@unmsm.edu.pe (J.L.R.); zsicchar@unmsm.edu.pe (R.S.-R.); rbritzke@unmsm.edu.pe (R.B.); maria.rivera2@unmsm.edu.pe (F.R.); noliverosr@unmsm.edu.pe (N.O.); 2 Museo de Historia Natural, Universidad Nacional Mayor de San Marcos, Av. Arenales 1256, Lima 15046, Peru; 3 Department of Natural History, NTNU University Museum, Norwegian University of Science and Technology, Erlings Skakkes gate 47B, 7012 Trondheim, Norway; jaime.g.m.lagos@ntnu.no (J.M.); 4 Department of Marine Zoology, Senckenberg Research Institute, 60325 Frankfurt am Main, Germany; andre.ampuero-leon@senckenberg.de (A.A.); 5 Department of Plant and Environmental Protection Services, University of Hawaii at Manoa, Honolulu, HI 96822, USA; congrain@hawaii.edu; 6 U.S. Department of Agriculture-Agricultural Research Service, Daniel K. Inouye U.S. Pacific Basin Agricultural Research Center, Tropical Pest Genetics and Molecular Biology Research Unit, Hilo, HI 96720, USA

**Keywords:** Ampullariidae, mitogenome, genomics, secondary structure, control region

## Abstract

Apple snails of the genus *Pomacea* Perry, 1810 (Mollusca: Caenogastropoda: Ampullariidae) are native to the Neotropics and exhibit high species diversity, holding cultural and ecological significance as an important protein source in Peru. However, most genetic studies in *Pomacea* have focused mostly on invasive species, especially in Southeast Asia, where they are considered important pests. In this study, we assembled and annotated the mitochondrial genomes of two *Pomacea* species native to the Peruvian Amazon: *Pomacea reevei* Ampuero & Ramírez, 2023 and *Pomacea aulanieri* (Deville & Hupé, 1850). The mitogenomes of *P. reevei* and *P. aulanieri* comprise 15,660 and 16,096 bp, respectively, and contain the typical 37 genes of the animal mitochondria with a large control region of 292 bp in *P. reevei* and 524 bp in *P. aulanieri*—which fall within the range of what is currently known in *Pomacea*. Comparisons with previously published mitogenomes in *Pomacea* revealed differences in the overlapping of adjacent genes, the size of certain protein-coding genes (PCGs) and the secondary structure of some tRNAs that are consistent with the phylogenetic relationships between these species. These findings provide valuable insights into the systematics and genomics of the genus *Pomacea*.

## 1. Introduction

The members of the genus *Pomacea* Perry, 1810 (Mollusca: Canogastropoda: Ampullariidae) are commonly known as apple snails and can be found in freshwater habitats such as small ponds, lakes, swamps and canals, extending throughout the Neotropical Region, from Argentina to southeast US and the Caribbean Islands [1]. However, in recent decades, certain species such as *Pomacea canaliculata* (Lamarck, 1822) or *Pomacea maculata* Perry, 1810 have gained growing importance as invasive species, becoming pests of high economic impact on natural habitats and agricultural areas, particularly in China and Southeast Asia [2].

Given the utility of mitochondrial DNA in elucidating animal systematics and evolutionary patterns [3,4,5], these studies have been primarily based on mitochondrial markers. Therefore, mitochondrial genomes have been made available for invasive species such as *P. canaliculata* [6,7], *P. maculata* [8] and *Pomacea diffusa* Blume, 1957 [9,10]. Comparative analyses between *Pomacea* mitochondrial genomes have provided new insights into their genetic diversity [11,12]. However, in order to gain a comprehensive understanding of the genus, we need to extend this type of study to native *Pomacea* species by sequencing and comparing their mitochondrial genomes.

Hayes et al. [13] identified four large clades within *Pomacea* based on three nuclear genes, as well as the mitochondrial markers *COI* and *16S rRNA*: the widespread *P. canaliculata* group, including similar species such as *P. maculata* [14] and *Pomacea occulta* Yang & Yu, 2019 [15]; the *Pomacea bridgesii* group, including *P. diffusa*; and the more restricted *Effusa* and *Flagellata* clades.

In the Peruvian Amazon, where apple snails are commonly referred to as “churos”, nearly 20 *Pomacea* species have been reported. However, our understanding of their taxonomy and evolutionary relationships remains limited [16], gaining more attention only in recent times [17,18,19]. While the phylogenetic relationships of certain Peruvian species such as *Pomacea aulanieri* (Deville & Hupé, 1850) of the *P. bridgesii* clade and the newly described *Pomacea reevei* Ampuero & Ramírez 2023 of the *P. canaliculata* clade have been recently elucidated by employing mitochondrial markers [18], the integration of mitochondrial genome information has the potential to offer fresh perspectives on their evolution and phylogenetics. Therefore, the aim of this study was to assemble and annotate the first mitochondrial genome of two native species of *Pomacea* from the Peruvian Amazon: *P. reevei* and *P. aulanieri*. 

## 2. Materials and Methods

### 2.1. Sample Collection and DNA Extraction

Samples of *P. reevei* and *P. aulanieri* were bought from local fishermen from Loreto, Peru (Napo and Huallaga basins, respectively). The identification of the specimens was based on shell morphology and anatomical characteristics and corroborated using molecular data [18]. Furthermore, *P. reevei* has recently been identified and described as a new species [19]. They were relaxed using ethanol, euthanized through thermal shock (4 °C) and then fixed and preserved in 96% ethanol [20]. Genomic DNA (gDNA) was extracted from 1 cm^3^ of tissue using the E.Z.N.A. Mollusc DNA Kit (OMEGA Bio-tek, Norcross, GA, USA). Concentration and quality of gDNA were measured using the Nanodrop Lite Spectrophotometer. DNA integrity was verified using 1% agarose gel electrophoresis. Voucher specimens (Figure S1) were deposited in the Museo de Historia Natural of the Universidad Nacional Mayor de San Marcos, Lima, Peru.

### 2.2. Genome Sequencing and Assembly

The whole genomes of *P. reevei* and *P. aulanieri* were sequenced by Macrogen Inc. (Seoul, South Korea) using an Illumina NovaSeq 6000 platform. Before sequencing, gDNA integrity was verified using TapeStation gDNA Screen Tape on the 4200 TapeStation System. Approximately 5 Gb of raw data from 150 bp paired-end reads were generated for each sample. Raw data quality was evaluated using FastQC (https://www.bioinformatics.babraham.ac.uk/projects/fastqc/, accessed on 15 October 2022) and processed using Fastp [21] by removing reads with >40% bases with Phred quality < Q15, trimming adapter sequences with >6 bases and trimming polyG tails. Mitogenome assemblies were obtained using GetOrganelle [22] with the *Pomacea canaliculata* mitochondrial genome (KU052865.1) as a starting reference. Bandage [23] was used to verify if the assembly graph was circular.

### 2.3. Annotation of Mitochondrial Genome and Predictions of Secondary Structures

The MITOS2 web server (http://mitos2.bioinf.uni-leipzig.de/index.py, accessed on 18 February 2023) [24] was used to predict the protein-coding genes (PCGs), transfer RNAs (tRNAs) and ribosomal RNAs (rRNAs). The annotation of the tRNAs was confirmed using ARWEN 1.2 (http://130.235.244.92/ARWEN/index.html, accessed on 18 February 2023) [25]. Protein-coding genes were manually corrected by searching for open reading frameworks (ORFs) with the NCBI tool ORFfinder (https://www.ncbi.nlm.nih.gov/orffinder/, accessed on 18 February 2023). Secondary structure of the RNAs was inferred using R2DT (https://rnacentral.org/r2dt, accessed on 19 February 2023) [26] and manually optimized using the *12S rRNA* Eukaryote reference model [27] and the *16S rRNA* Mollusca and *Toxoplasma gondii* (Nicolle & Manceaux, 1908) reference models [28,29]. Potential secondary structure of the control region (CR) was predicted using the RNAfold web server (http://rna.tbi.univie.ac.at//cgi-bin/RNAWebSuite/RNAfold.cgi, accessed on 19 February 2023). All secondary structures were drawn using Inkscape 1.1 (http://www.inkscape.org/, accessed on 25 February 2023). The mitochondrial genome maps of both species were drawn using the CGView tools [30] available at the web server Proksee (https://proksee.ca/, accessed on 26 February 2023). The mitochondrial genome and annotation files of *P. canaliculata* (KJ739609.1), *P. diffusa* (MF373586.1), *P. maculata* (MF401379.1) and *P. occulta* (KR350466.1) were retrieved from GenBank. We performed a re-annotation of these mitochondrial genomes using MITOS2 Web Server [24] and ORFfinder to verify current annotations.

### 2.4. Comparative Analysis

Nucleotide composition was calculated using MEGA X [31] for complete mitogenomes, PCGs, tRNAs, rRNAs and CR. Compositional asymmetry was calculated using the formulas for AT-skew = (A − T)/(A + T) and GC-skew = (G − C)/(G + C) [32]. Relative synonymous codon usage (RSCU) values were calculated using MEGA X [31]. Nucleotide diversity (π) was determined using DnaSP6 [33] with a sliding window analysis of 200 bp and a step size of 25 bp. Overall mean p-distance was calculated for each PCG using MEGA X [31]. The nonsynonymous nucleotide substitutions per nonsynonymous site (Ka), the synonymous nucleotide substitutions per synonymous site (Ks) and the ratio of nucleotide nonsynonymous to synonymous substitutions (Ka/Ks) were calculated for each PCG using DnaSP6 [33].

### 2.5. Phylogenetic Analysis

The phylogenetic analyses included 35 sequences of Caenogastropoda species and two outgroup species: *Haliotis rubra* Leach, 1814 (AY588938) and *Aplysia californica* Cooper, 1863 (AY569552). Orthologs of the nucleotide sequences of the 13 PCGs, *12S rRNA* and *16S rRNA* were individually aligned using Muscle 3.8 [34] implemented in Aliview 1.27 [35]. Ambiguous aligned regions were identified and removed using Gblocks 0.91b [36] under relaxed conditions in PhyloSuite v1.2.3 [37]. Individual gene alignments were concatenated using PhyloSuite v1.2.3 [37]. PartitionFinder2 v2.1.1 [38] in PhyloSuite v1.2.3 [37] was used to determine the best partitioning scheme and substitution model. Maximum Likelihood (ML) analysis was performed using IQ-TREE 2.1.2 [39] on the CIPRES Science Gateway web server [40], with 10,000 ultrafast bootstrap replicates. Bayesian Inference (BI) analysis was performed using MrBayes 3.2 [41] on the CIPRES Science Gateway web server [40], with two independent runs of four Markov Chain Monte Carlo (MCMC) chains running for 2 million generations and sampling every 1000 generations with a burn-in of 25%. The resulting phylogenetic trees were visualized through FigTree v1.4.3 [42] and edited with Inskape 1.1. (http://www.inkscape.org/, accessed on 25 March 2023).

## 3. Results and Discussion

### 3.1. Organization and Structure of the Mitogenomes

**Figure 1:**
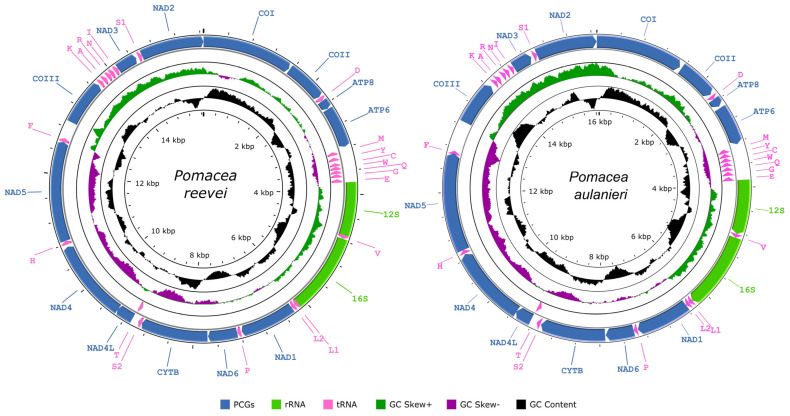
Graphical map of the mitochondrial genomes of *Pomacea reevei* and *Pomacea aulanieri*.

The mitochondrial genomes of *P. reevei* and *P. aulanieri* (GenBank accession numbers OR253802 and OR253803, respectively) contained the typical 37 genes (13 PCGs, two rRNAs and 22 tRNAs) and a large non-coding region known as the CR (Figure 1, Table 1 and Table 2), although circular assemblies were not achieved, due to repetitive sequences within this region. Previous studies in *Pomacea* also failed to recover the complete CR [12] or have not reported it [11]. Most genes, including all PCGs and rRNAs, were encoded on the heavy strand (H-strand), whereas eight tRNAs (*tRNA-Met, tRNA-Tyr, tRNA-Cys, tRNA-Trp, tRNA-Gln, tRNA-Gly, tRNA-Glu, tRNA-Thr*) were located on the light strand (L-strand). The lengths of both mitogenomes were similar: 15,660 bp in *P. reevei* and 16,096 bp in *P. aulanieri*. These values closely matched previous reports in other *Pomacea* species, where lengths between 15,516 (*P. occulta*) and 16,373 bp (*P. diffusa*) have been reported [6,7,8,9,10,11,12]. Both mitogenomes showed identical gene arrangements, which are consistent with other Ampullaridae species. Including the CR, 24 intergenic regions were identified in *P. reevei*, ranging from 2 to 292 bp, for a total of 714 bp (4.6% of mitogenome), whereas 25 intergenic regions were identified in *P. aulanieri*, ranging from 3 to 524, for a total of 1081 bp (6.7% of mitogenome). The intergenic regions represented 4.8% of the mitogenome in *P. canaliculata* [6], 3.5% in *P. maculata* [12] and 10.1% in *P. diffusa* [12].

The longest overlapping region was found between *NAD5/tRNA-Phe* in *P. aulanieri* (21 bp), although this overlapping was only 2 bp in *P. reevei*. This overlapping has also been reported in *P. diffusa* [11], with a similar length (20 bp) to *P. aulanieri,* but not in *P. canaliculata*, *P. maculata* nor *P. occulta* [11,12]. Both *P. reevei* and *P. aulanieri* also showed an overlapping region of 7 bp between *NAD4L/NAD4* that has been reported in other Ampullariidae such as *Marisa cornuarietis* (Linnaeus, 1758) [43] and *P. diffusa* [11], as well as other Caenogastropoda [44,45,46]. The re-annotation of the mitogenomes of *P. canaliculata*, *P. maculata* and *P. occulta* in the present study showed that these species also had this overlapping, although it had not been previously reported [6,7,11,12]. *P. aulanieri* additionally showed two small overlapping regions between *tRNA-Pro/NAD6* (1 bp) and *tRNA-Ser1/NAD2* (1 bp). The overlapping between *NAD1*/*tRNA-Pro* reported for *P. canaliculata*, *P. maculata* and *P. occulta* [6,7,11,12] has not been found in *P. reevei* nor *P. aulanieri*. The protein-coding genes, rRNAs and tRNAs comprised 71.78%, 14.37% and 9.36% of the whole mitochondrial genome of *P. reevei*, respectively. Similar values were observed in *P. aulanieri*, where protein-coding genes, rRNAs and tRNAs represented 69.93%, 14.34% and 9.23%, respectively.

### 3.2. Nucleotide Composition

The base composition of the mitogenomes of *P. reevei* (30.10% A, 44.88% T, 13.14% C, 15.87% G) and *P. aulanieri* (29.48% A, 39.37% T, 14.83% C, 16.32% G) was similar (Table 3), showing a high content of A + T (70.98% in *P. reevei* and 68.85% in *P. aulanieri*), a negative AT-skew (−0.15 in *P. reevei* and −0.14 in *P. aulanieri*) and a positive GC-skew (0.09 in *P. reevei* and 0.05 in *P. aulanieri*). Comparing the different sets of elements of the mitogenome, the CR (77.4%) showed the highest A + T content in *P. reevei*, whereas in *P. aulanieri*, the tRNAs (71.58%) and the CR (71.56%) showed the highest values. In both mitogenomes, the lowest A+T content was found in the PCGs. The PCGs also showed the lowest AT-skew, whereas the rRNAs had the highest AT-skew in both mitogenomes. Interestingly, although the GC-skew values were positive for the whole mitogenome, PCGs, rRNAs and tRNAs of *P. reevei* and *P. aulanieri*, the CR of the former showed a positive value, whereas in the latter, this region had a negative value. This difference in the GC-skew of the CR can also be observed in Figure 1.

**Figure 2:**
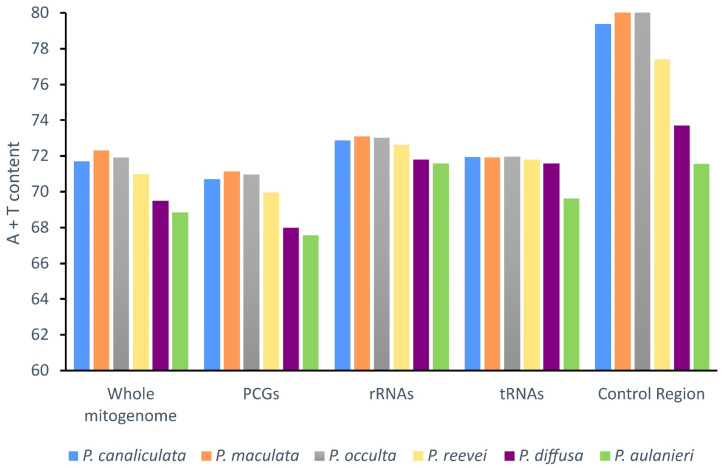
A + T content of the mitochondrial genome of *P. aulanieri*, *P. canaliculata*, *P. diffusa*, *P. maculata*, *P. occulta* and *P. reevei*. (PCGs: Protein-Coding Genes).

The comparison of the A + T content (Figure 2), between *P. reevei*, *P. aulanieri* and other species of *Pomacea*, showed that the A + T content of *P. reevei* (70.98%) was similar to species such as *P. canaliculata* (71.69%), *P. maculata* (72.31%) and *P. occulta* (71.93%), whereas the A+T content of *P. aulanieri* (68.85%) was the lowest among the compared species and similar to *P. diffusa* (69.49%). A similar pattern was found after comparing the A+T content of the PCGs, rRNAs, tRNAs and especially the CR, where *P. reevei* (77.40%), *P. canaliculata* (79.39%), *P. maculata* (85.82%) and *P. occulta* (86.57%) of the *P. canaliculata* clade showed high values, in contrast to *P. aulanieri* (71.56%) and *P. diffusa* (73.70%) of the *P. bridgesii* clade, although these values could be affected by the recovered length of the CR in each species.

**Figure 3:**
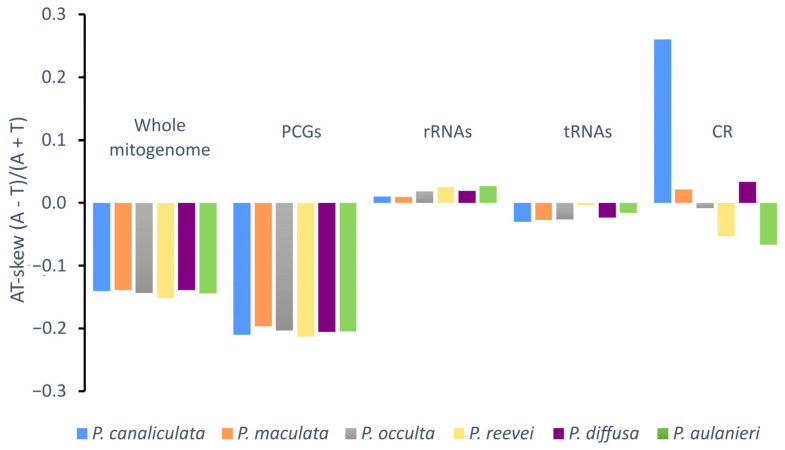
AT-skew of the mitochondrial genome of *P. aulanieri*, *P. canaliculata*, *P. diffusa*, *P. maculata*, *P. occulta* and *P. reevei*. (PCGs: Protein-Coding Genes).

**Figure 4:**
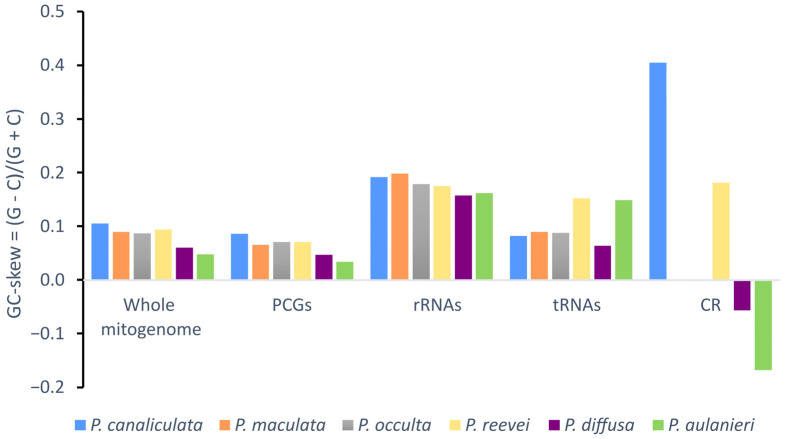
GC-skew of the mitochondrial genome of *P. aulanieri*, *P. canaliculata*, *P. diffusa*, *P. maculata*, *P. occulta* and *P. reevei*. (PCGs: Protein-Coding Genes).

Negative AT-skew values were observed for the whole mitogenome, protein-coding genes and tRNAs of all *Pomacea*, whereas positive values were observed in the rRNAs (Figure 3). Although the CR of *P. reevei* and *P. aulanieri* showed a negative AT-skew, in other species, this value was positive (e.g., *P. canaliculata* or *P. diffusa*). A similar pattern was observed in the GC-skew (Figure 4), where the whole mitogenomes and most of the features (PCGs, rRNAs and tRNAs) showed positive values, except for the CR of *P. aulanieri* and *P. diffusa*. Perna and Kocher [32] suggested that differences in the number and direction of AT-skew and GC-skew values reflect variations in selective pressures and nucleotide substitution processes. Additionally, the positive AT-skew values of rRNAs and slightly negative values for tRNAs contrast with the strongly negative values of PCGs. Therefore, this finding may be a signal of the strong selective pressures that force PCGs to eliminate deleterious mutations.

### 3.3. Protein-Coding Genes and Codon Usage

The length of the PCGs ranged from 159 bp (*ATP8*) to 1707 bp (*NAD5*) in *P. reevei* and the same was observed in *P. aulanieri*, but *NAD5* was slightly longer (1728 bp). A comparison of the length of all PCGs among reported *Pomacea* mitogenomes [6,7,8,9,10,11,12] revealed that the size of *COI* (1536 bp), *COII* (687 bp), *ATP8* (159 bp), *NAD6* (495 bp), *CytB* (1140 bp), *NAD4L* (297 bp), *COIII* (780 bp) and *NAD3* (354 bp) is conserved among all species. The *ATP6* of most *Pomacea* showed the same length (699 bp), with the exception of *P. diffusa* (714 bp). In *NAD1*, *NAD4* and *NAD5,* we identified three groups: (1) *P. reevei*, with 939, 1374 and 1707 bp; (2) *P. maculata*, *P. canaliculata* and *P. occulta,* with 960, 1368 and 1710 bp; and (3) *P. aulanieri* and *P. diffusa*, with 945, 1362 and 1728 bp, respectively. The most conspicuous variation in size was found in *NAD2:* 1062 bp (*P. canaliculata* and *P. maculata*), 1065 bp (*P. occulta*), 1071 bp (*P. diffusa*) and 1074 bp (*P. reevei* and *P. aulanieri*).

The start codon in all PCGs of both species was the same (ATG), similar to reports from *P. diffusa* [9] and *Marisa cornuarietis* [43], although in *P. canaliculata*, *P. maculata* and *P. occulta,* the start codon of *COIII* was ATA. The most common stop codon was TAA, found in 12 proteins of *P. reevei* and 9 of *P. aulanieri*. Only *ATP6* in *P. reevei* used TAG, whereas *ATP8*, *ATP6*, *NAD3* and *NAD2* ended with this stop codon in *P. aulanieri*. Most *Pomacea* species also showed a preference for TAA as a stop codon [11,12].

**Figure 5:**
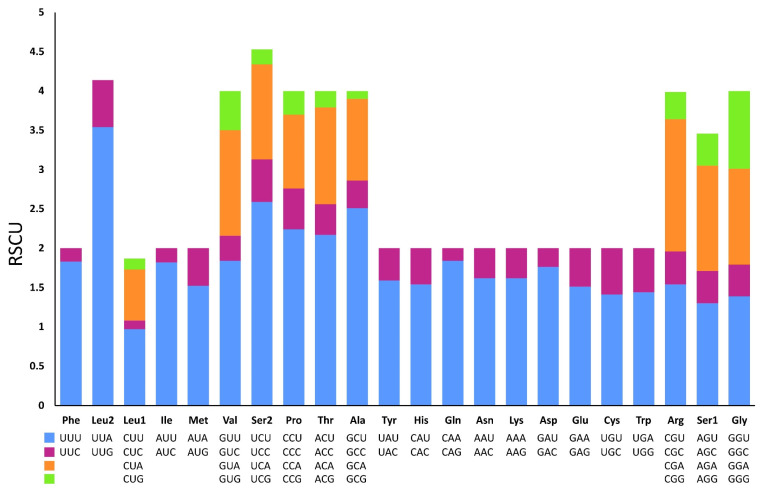
Relative synonymous codon usages (RSCU) in the mitogenome of *P. reevei*.

Excluding the stop codons, 3734 and 3739 codons were identified in the mitogenome of *P. reevei* and *P. aulanieri*, respectively. The codon usage pattern analysis of the PCGs in *P. reevei* (Table 4, Figure 5) showed that the most frequent amino acids were Leu (16.6%), Ser (14.18%) and Phe (9.1%), and the least used were Cys (1.18%) and Arg (1.5%). The most frequently detected codons in *P. reevei* were UUA (Leu2) and UUU (Phe), whereas the least common codon was CGG (Arg). Similar values were observed in *P. aulanieri* (Table S1, Figure S2), although here, CGC (Arg) was the least frequently used codon. In both species, all amino acids have their preferred codons.

Sun et al. [47] indicated that nucleotide compositional asymmetry in the coding strand could affect codon and amino acid usage. Therefore, we expected that based on the negative AT-skew and positive GC-skew of the *Pomacea* mitogenome, amino acids coded by GT-rich codons might be more frequently used than AC-rich codons. We corroborated this in both species (*P. reevei* and *P. aulanieri*), since the most abundant amino acids were Leu2 (TTN) and Phe (TTN). Yang et al. [11] found that in *Pomacea*, codons ending in A or T were more frequent than those ending in C or G. This pattern was found in this study, since in *P. reevei* and *P. aulanieri* codons ending in T (47.2% and 43.9%, respectively) and A (35.4% and 33.3%, respectively) collectively represent more than half of all codons. 

### 3.4. Ribosomal and Transfer RNA Genes

Similar to other *Pomacea* species, the mitochondrial genome of *P. reevei* and *P. aulanieri* contained two ribosomal RNA. The *12S rRNA* had a length of 909 bp in *P. reevei* and 960 bp in *P. aulanieri*, whereas the length of *16S rRNA* was 1340 bp in *P. reevei* and 1348 bp in *P. aulanieri*. Based on the *12S rRNA* and *16S rRNA* length in *Pomacea*, three groups can be identified: (1) *P. reevei*, with a length of 909 and 1340 bp, respectively; (2) *P. maculata*, *P. canaliculata* and *P. occulta,* with a range of lengths of 929–936 and 1331–1336 bp, respectively; and (3) *P. aulanieri* and *P. diffusa*, with a range of lengths of 952–958 and 1346–1348 bp, respectively. 

**Figure 6:**
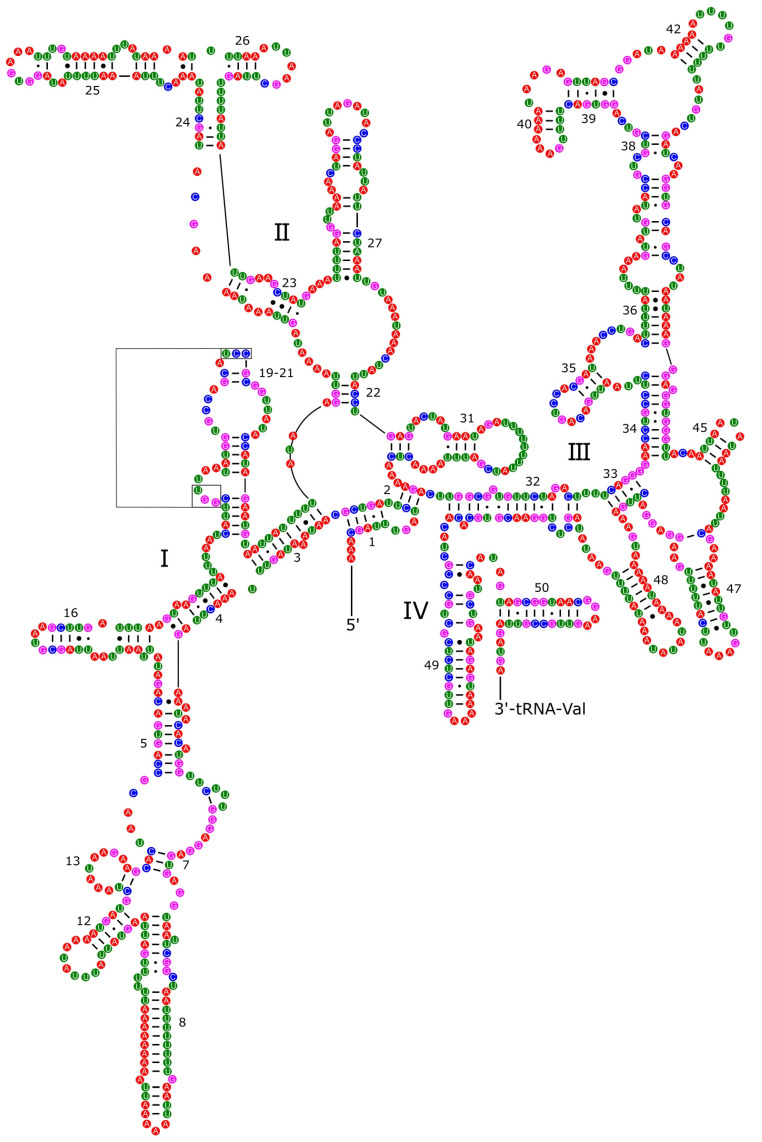
Secondary structure of the *12S rRNA* of *P. reevei.* Domains are indicated with Roman numbers and helices are numbered following Hickson et al. [27].

Secondary structures of ribosomal RNAs have been poorly explored among mollusks and the structures presented here for *P. reevei* and *P. aulanieri* are the first inferred within *Pomacea*. The secondary structure of the *12S rRNA* of *P. reevei* (Figure 6) and *P. aulanieri* (Figure S3) had an identical organization of four structural domains, with Domain III as the most conserved region, allowing its successful PCR across a wide variety of taxa [48]. Here, we found that the structure of Domain III in *P. reevei* and *P. aulanieri* is similar to previous reports in other mollusks [27,49,50,51]. On the other hand, Domains I and II have been poorly studied and they were difficult to infer in both species. Simon et al. [48] indicated that these regions showed less homoplasy than Domains III and IV so they could be more phylogenetically informative and particularly useful for resolving ancient evolutionary relationships.

**Figure 7:**
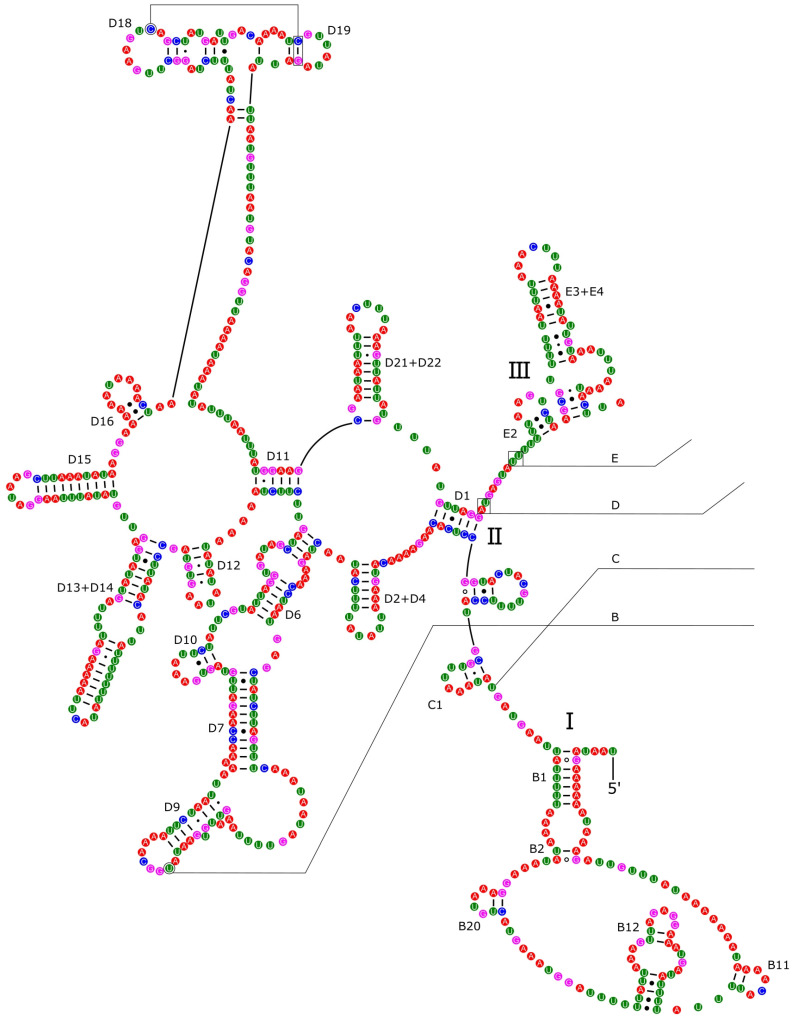
Secondary structure of the I–III domains of the *16S rRNA* of *P. reevei.* Domains are indicated with Roman numbers and helices are numbered following Wuys et al. [29].

**Figure 8:**
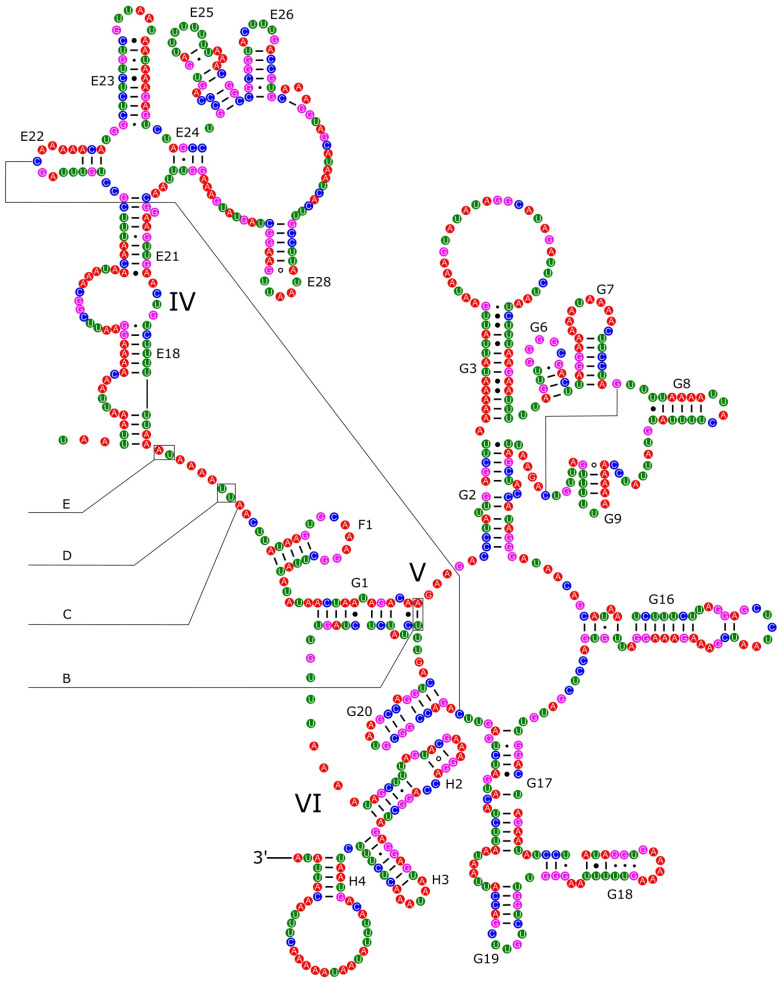
Secondary structure of the IV–VI domains of the *16S rRNA* of *P. reevei.* Domains are indicated with Roman numbers and helices are numbered following Wuys et al. [29].

The *16S rRNA* had a length of 1340 bp in *P. reevei* (Figure 7 and Figure 8) and 1348 bp in *P. aulanieri* (Figures S4 and S5), showing the same organization, with six structural domains. These secondary structures agree with the mollusk consensus structure proposed by Lydeard et al. [28], and they have the same number of stems and loops as the models inferred for the Caenogastropoda *Cacozeliana lacertina* (now *Cacozeliana granarium* (Kiener, 1841)) and *Paracrostoma paludiformis* (currently *Brotia paludiformis* (Solem, 1966)) (available at https://crw-site.chemistry.gatech.edu/, accessed on 10 March 2023). Smith and Bond [52] observed that among spiders, the secondary structure of *16S rRNA* could be phylogenetically useful at higher taxonomic levels, whereas the loops, as the most variable regions, could be used at family level relationships. No study has evaluated the phylogenetic signal of the secondary structure of the *16S rRNA* among Caenogastropoda.

The length of the tRNAs varied from 62 to 73 bp in *P. reevei* (Table 1), and from 62 to 74 bp in *P. aulanieri* (Table 2), a wider range than in *P. canaliculata, P. maculata* and *P. occulta* (64–70 bp), but smaller than in *P. diffusa* (62–76 bp). Comparing the length variation along the whole set of 22 tRNAs in *Pomacea*, only *tRNA-Asp* had the same length in all species, whereas the most variable tRNAs were *tRNA-Ile* (70–76 bp), *tRNA-Ala* (68–74 bp) and *tRNA-Thr* (66–71 bp).

**Figure 9:**
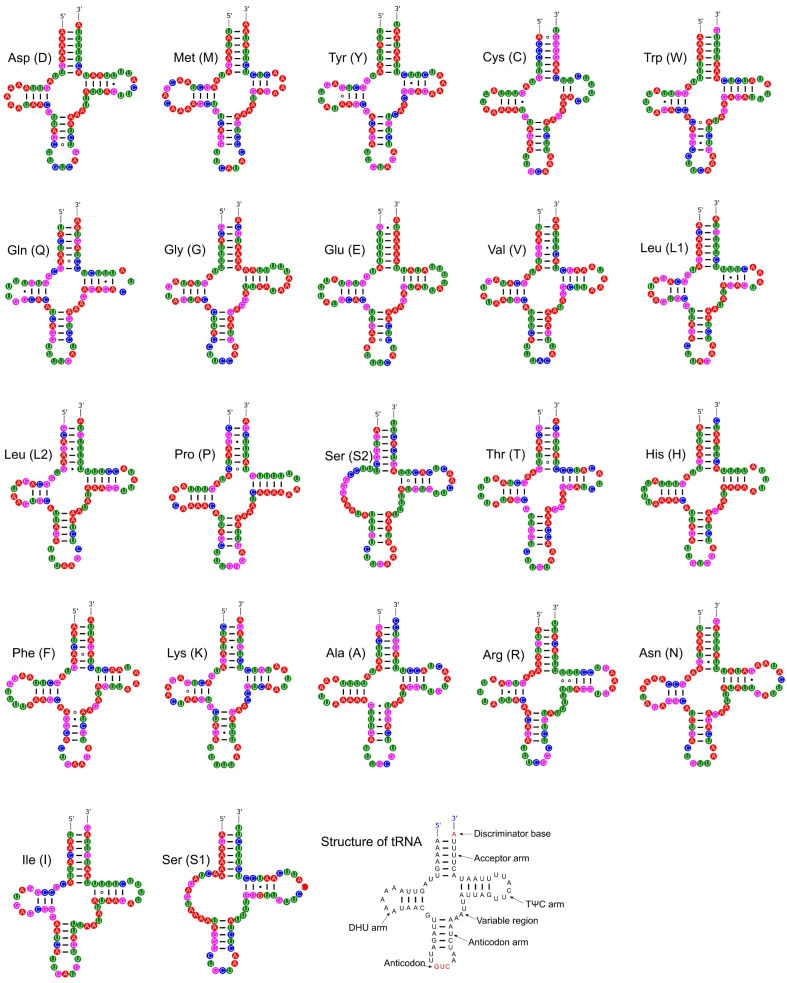
Secondary structures of the tRNA genes in the mitogenome of *P. reevei.*

Most tRNAs showed the typical clover leaf secondary structure, with four arms and loops. However, both tRNA for Serine in *P. reevei* showed a large loop instead of the typical stem-loop structure in the DHU domain (Figure 9), whereas in *P. aulanieri,* only *tRNA-Ser2* showed this loop (Figure S6). Yang et al. [12] reported a similar loop in the *tRNA-Ser1* of *P. maculata*, but Yang et al. [11] indicated that all tRNAs in *P. canaliculata*, *P. maculata* and *P. diffusa* had the typical clover leaf structure. However, after inferring the secondary structure of the tRNA of *P. canaliculata* (KJ739609.1), *P. maculata* (MF401379.1), *P. occulta* (KR350466.1) and *P. diffusa* (MF373586.1) using ARWEN 1.2 [25], we found that in the first three species, the *tRNA-Ser1* had a loop in the DHU domain, whereas in *P. diffusa*, *tRNA-Ser2* had a loop in the same domain. Therefore, both *P. aulanieri* and *P. diffusa* of the *P. bridgesii* clade had a loop in *tRNA-Ser2*, whereas *P. reevei*, *P. canaliculata*, *P. maculata* and *P. occulta* of the *P. canaliculata* clade had a loop in *tRNA-Ser1,* although *P. reevei* also had a loop in *tRNA-Ser2.*

### 3.5. Control Region

The largest intergenic region of the mitochondrial genome of both species was located between *tRNA-Phe* and *COIII* and showed all the specific characteristics for the CR [53,54]: (i) represents the longest intergenic region; (ii) shows a high A+T content; (iii) has a potential secondary structure; and (iv) contains repetitive elements. We retrieved a CR length of 294 bp in *P. reevei* and 524 bp in *P. aulanieri*. Both are within the reported range in *Pomacea*, from 141 bp (*P. occulta*) to 806 bp (*P. diffusa*). In another Ampullariid, *Marisa cornuarietis*, this region is considerably smaller (63 bp) [43]. Brauer et al. [54] also found interspecific differences in the CR length of *Conus*, with the size of this region in *Conus consors* Sowerby I, 1833 (698 bp) being five-fold longer than in *Conus textile* Linnaeus, 1758 or *Conus borgesi* Trovão, 1979. The CR in *Conus quercinus* Lightfoot, 1786 is even longer (943 bp) [46]. Within *Pomacea*, species from the *P. bridgesii* clade (e.g., *P. aulanieri*) seem to have a longer CR than those of the *P. canaliculata* clade (e.g., *P. reevei*).

A repetitive unit of 12 bp (ACATACATACAT) was manually identified in the CR of *P. reevei*, with eight repetitions on the H-strand and three repetitions on the L-strand. A repeat unit was also found in the CR of *P. aulanieri*, with a length of 23 bp (ATATAATCTCTATATGTGTATGG) and six copies on the H-strand. Different repetitive units have been reported in *Pomacea*: 5 copies of GATACTATAATATAAA (16 bp) in *P. occulta* [8]; 13 copies on the H-strand and 11 repeats on the L-strand of AAGATACTATAATATA (16 bp) in *P. maculata* [12]; 11 repeats of TAAGATATAAAGAAACTAAGAGA (23 bp) in *P. canaliculata* [7]; and 19 copies on the H-strand and 18 repeats on the L-strand of ATCTATACATAC (12 bp) in *P. diffusa* [9]. Maynard et al. [55] found many AT repeats in the *Haliotis rubra* mitogenome, suggesting that if the number of AT repeats were polymorphic, it could become a marker for individual typing. The variations observed in the number and motif of the repetitive units of the mitochondrial CR of *Pomacea* suggest that it would be valuable to evaluate alternative strategies to resolve this repetitive region, such as long read sequencing [56].

**Figure 10:**
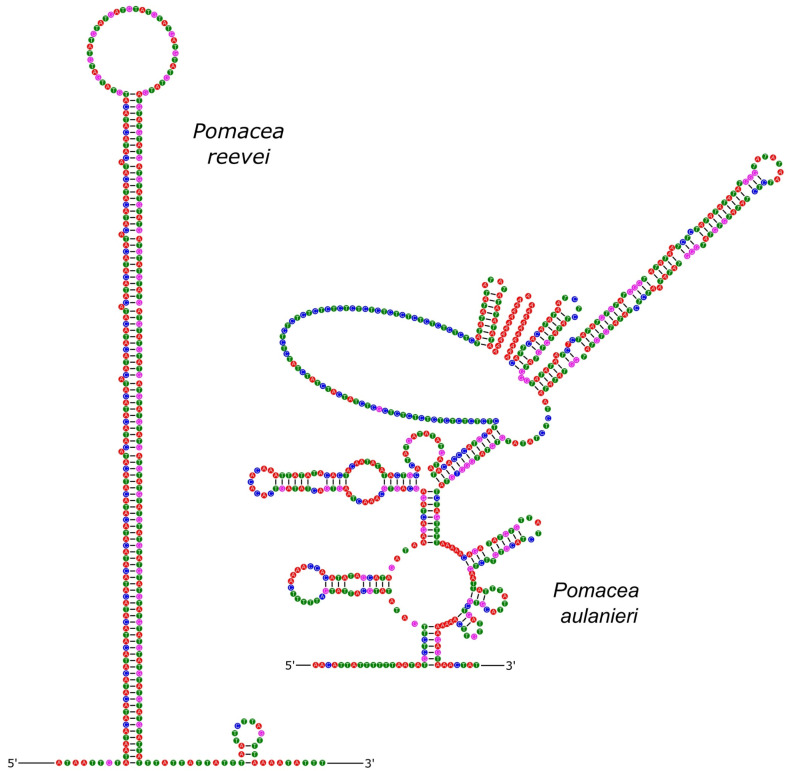
Secondary structure of the control region of *P. reevei* and *P. aulanieri*.

The inferred secondary structure of the CR of *P. reevei* and *P. aulanieri* showed differences in the number and organization of stems and loops between both species (Figure 10). Similar differences have been found between the CR of *Conus consors*, *C. borgesi* and *C. textile* [54]. It has been suggested that stem-loop structures may start replication in animal mitochondria [57], but they have been poorly studied in molluscan mitochondrial genomes. 

### 3.6. Genetic Variation among Pomacea

**Figure 11:**
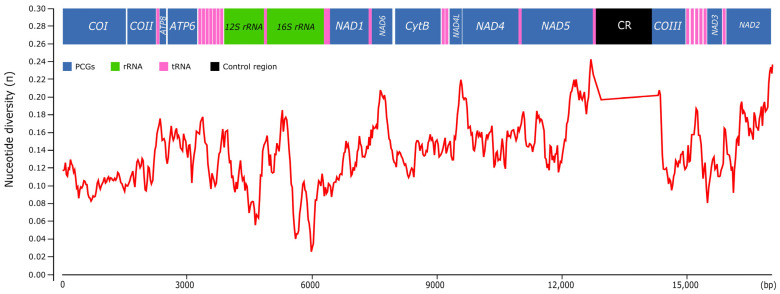
Nucleotide diversity (π) among the *Pomacea* mitogenomes available in GenBank and the mitogenomes included in this study.

The plot of sequence variation exhibited a variable nucleotide diversity along *Pomacea* mitogenomes (Figure 11). The overall mean p-distance calculated for each protein-coding gene ranged from 0.127 (*COI*) to 0.244 (*ATP8*), with other genes such as *NAD2* (0.213), *NAD6* (0.206), *NAD5* (0.198) or *NAD1* (0.190) also showing elevated values. In contrast, the ribosomal RNAs showed lower variability values: 0.139 (*12S rRNA*) and 0.136 (*16S rRNA*), whereas the CR displayed the highest value of the whole mitogenome (0.435). Castellana et al. [58] found among vertebrates that *COI, COII* and *COIII* were the most conserved mitochondrial genes, contrasting with the *ATP8* and *NAD* genes that were the most variable, whereas *CytB* and *ATP6* showed intermediate values. Peretolchina et al. [59] compared four mitogenomes among Caenogastropoda family Baicaliidae and found little variability among *COI*, recommending instead *COII* and *COIII* for phylogenetic studies and *CytB* and *NAD* genes for population studies. Here, in *Pomacea*, we found similar patterns, with *ATP8*, *CytB* and *NAD* genes being the most variable, and *COI, COII* and *COIII* as the most conserved. Fonseca et al. [60] suggested that the phylogenies inferred from *NAD5* are the most topologically concordant with the one produced by the whole mitogenome. Although we have not tested this for *Pomacea*, the high variability of this gene suggests it could be useful for phylogenetic studies, along with other genes such as *CytB* or *NAD1*. We found in *Pomacea* that all PCGs had Ka/Ks values below 1 (Figure S7), indicating that they were evolved under purifying selection. Among the 13 PCGs, *COI* showed the slowest evolutionary rate, whereas *NAD6* had the fastest rate, which is concordant with a previous study in the three invasive *Pomacea* species [11]. 

**Figure 12:**
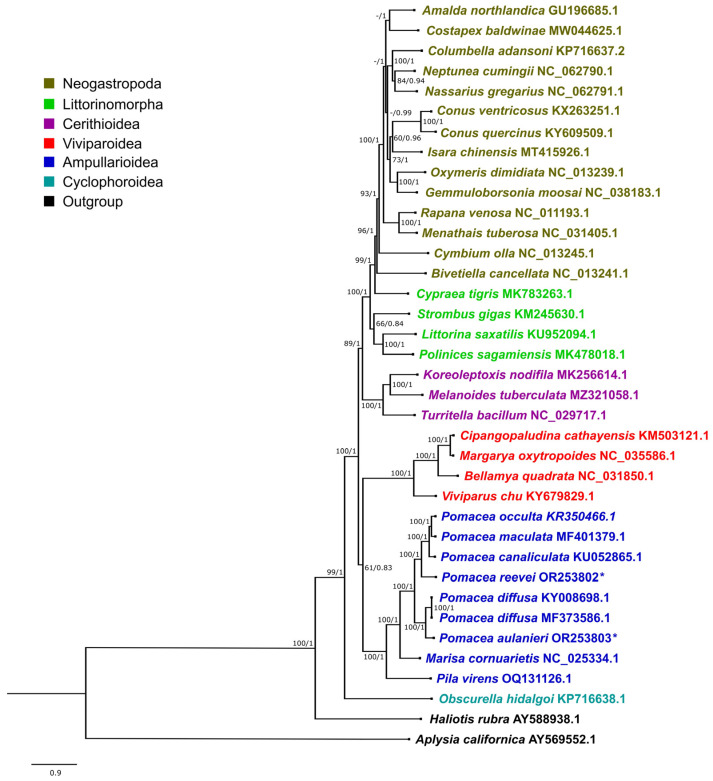
Phylogenetic relationships of Caenogastropoda species based on protein-coding genes and ribosomal RNAs, inferred with Bayesian Inference. Numbers on branches are Maximum Likelihood bootstrap values (**left**) and posterior probabilities from BI (**right**). *Pomacea* species sequenced in this study are marked with an asterisk.

In this study, we have found many differences in the mitogenome structure in *Pomacea* such as the presence and length of overlapping regions, PCG lengths, COIII start codons, tRNA length range, secondary structures of tRNAs for Serine and ribosomal RNA lengths, summarized in Table 5. These differences are concordant with the topology of the molecular phylogeny (Figure 12), although *P. reevei* of the *P. canaliculata* clade showed some similarities to *P. bridgesii* clade species such as *P. aulanieri*. 

### 3.7. Phylogenetic Analysis

The phylogenetic trees recovered using Maximum Likelihood (ML) and Bayesian Inference (BI) showed an almost identical topology, except for *Costapex baldwinae* Harasewych, Uribe & Fedosov, 2020 within Neogastropoda (Figure 12 and Figure S8). The inference generated with BI exhibited higher support values than ML, and it was used to represent the evolutionary relationships of Caenogastropoda (Figure 12), whereas the ML tree is shown in Figure S8. Most clades on Figure 12 showed a high support (BI: 1, ML: >90), with the exception of Ampullarioidea + Viviparoidea (BI: 0.83, ML:61), which have been classified with *Obscurella hidalgoi* (Crosse, 1864) (Cyclophoroidea) within Architaenioglossa, although the monophyly of this group has not been recovered using mitogenomes [61]. 

Although mitogenome sequence KY008698.1 is identified in GenBank as *P. bridgesii* (Reeve, 1856), it was published as *P. diffusa* [10] and posteriorly analyzed with this species name [11,12]. We compared this sequence with *P. diffusa* MF373586.1 and found that the only difference was on the sizes of the CR, being longer in KY008698.1 due to the higher number of repetitive units. Therefore, we concluded that both sequences represent the mitogenome of *P. diffusa*.

The phylogenetic relationships within Ampullariidae were recovered with the highest support values, both in the BI and ML trees, with *Pomacea* recovered as monophyletic. Within *Pomacea*, we recovered two main clades: (1) *P. canaliculata, P. maculata, P. occulta* and *P. reevei*; and (2) *P. diffusa* and *P. aulanieri*. Hayes et al. [13] identified four groups within *Pomacea* based on nuclear and mitochondrial markers: (1) *P. canaliculata* clade, (2) *P. bridgesii* clade, (3) *Effusa* clade and (4) *Flagellata* clade. Posteriorly, *P. maculata* was differentiated within the *P. canaliculata* clade [14], and Ramírez et al. [18] recovered *P. reevei* (as *Pomacea* sp. 1) within the *P. canaliculata* clade and *P. aulanieri* within the *P. bridgesii* clade. These phylogenetic relationships are confirmed here based on mitochondrial genomes, and agree with previous results [11,12]. So far, recovered *Pomacea* mitogenomes have been restricted to the *P. canaliculata* and *P. bridgesii* clades, but no mitogenome is available for the *Effusa* or *Flagellata* clade.

## 4. Conclusions

Mitochondrial genomes in *Pomacea* are highly variable compared to other snail genera. We found striking variations in CR length between *P. reevei* and *P. aulanieri*. Moreover, compared with previously published mitogenomes, we found differences in the organization and structure of features such as the PCGs and tRNAs, consistent with the phylogenetic relationships between the *P. canaliculata* and *P. bridgesii* clades. A similar comparison with other *Pomacea* clades, as well as other Ampullariidae genera, would provide new insights into the evolution of these freshwater snails.

We show that comparative analysis of mitochondrial sequences can help us to better understand the evolutionary relationships within the *Pomacea* genus. Thus, we want to highlight the importance of generating more whole-genome sequence data in order to represent the remaining *Pomacea* species. 

## Figures and Tables

**Table 1:** Organization of the mitochondrial genome of *P. reevei*.

Gene	Strand	Position	Size (bp)	Intergenic Length	Anticodon	Start Codon	Stop Codon
*COI*	H	1–1536	1536	18	-	ATG	TAA
*COII*	H	1555–2241	687	14	-	ATG	TAA
*tRNA-Asp*	H	2256–2323	68	0	GTC	-	-
*ATP8*	H	2324–2482	159	8	-	ATG	TAA
*ATP6*	H	2491–3189	699	33	-	ATG	TAG
*tRNA-Met*	L	3223–3288	66	39	CAT	-	-
*tRNA-Tyr*	L	3328–3393	66	38	GTA	-	-
*tRNA-Cys*	L	3432–3493	62	3	GCA	-	-
*tRNA-Trp*	L	3497–3562	66	8	TCA	-	-
*tRNA-Gln*	L	3571–3633	63	5	TTG	-	-
*tRNA-Gly*	L	3639–3704	66	26	TCC	-	-
*tRNA-Glu*	L	3731–3797	67	0	TTC	-	-
*12S rRNA*	H	3798–4706	909	0	-	-	-
*tRNA-Val*	H	4707–4772	66	0	TAC	-	-
*16S rRNA*	H	4773–6112	1340	0	-	-	-
*tRNA-Leu1*	H	6113–6175	63	0	TAG	-	-
*tRNA-Leu2*	H	6176–6241	66	0	TAA	-	-
*NAD1*	H	6242–7180	939	13	-	ATG	TAA
*tRNA-Pro*	H	7194–7260	67	0	TGG	-	-
*NAD6*	H	7261–7755	495	10	-	ATG	TAA
*CytB*	H	7766–8905	1140	3	-	ATG	TAA
*tRNA-Ser2*	H	8909–8973	65	35	TGA	-	-
*tRNA-Thr*	L	9009–9074	66	7	TGT	-	-
*NAD4L*	H	9082–9378	297	−7	-	ATG	TAA
*NAD4*	H	9372–10,745	1374	38	-	ATG	TAA
*tRNA-His*	H	10,784–10,849	66	0	GTG	-	-
*NAD5*	H	10,850–12,556	1707	−2	-	ATG	TAA
*tRNA-Phe*	H	12,555–12,622	68	0	GAA	-	-
*CR*	-	12,623–12,914	292	0	-	-	-
*COIII*	H	12,915–13,694	780	27	-	ATG	TAA
*tRNA-Lys*	H	13,722–13,787	66	17	TTT	-	-
*tRNA-Ala*	H	13,805–13,872	68	21	TGC	-	-
*tRNA-Arg*	H	13,894–13,963	70	2	TCG	-	-
*tRNA-Asn*	H	13,966–14,038	73	15	GTT	-	-
*tRNA-Ile*	H	14,054–14,123	70	0	GAT	-	-
*NAD3*	H	14,124–14,477	354	40	-	ATG	TAA
*tRNA-Ser1*	H	14,518–14,584	67	0	GCT	-	-
*vNAD2*	H	14,585–15,658	1074	2	-	ATG	TAA

**Table 2:** Organization of the mitochondrial genome of *P. aulanieri*.

Gene	Strand	Position	Size (bp)	Intergenic Length	Anticodon	Start Codon	Stop Codon
*COI*	H	1–1536	1536	35	-	ATG	TAA
*COII*	H	1572–2258	687	27	-	ATG	TAA
*tRNA-Asp*	H	2286–2353	68	0	GTC	-	-
*ATP8*	H	2354–2512	159	30	-	ATG	TAG
*ATP6*	H	2543–3241	699	28	-	ATG	TAG
*tRNA-Met*	L	3270–3332	63	21	CAT	-	-
*tRNA-Tyr*	L	3354–3418	65	22	GTA	-	-
*tRNA-Cys*	L	3441–3502	62	22	GCA	-	-
*tRNA-Trp*	L	3525–3590	66	25	TCA	-	-
*tRNA-Gln*	L	3616–3681	66	26	TTG	-	-
*tRNA-Gly*	L	3708–3774	67	34	TCC	-	-
*tRNA-Glu*	L	3809–3873	65	0	TTC	-	-
*12S rRNA*	H	3874–4833	960	0	-	-	-
*tRNA-Val*	H	4834–4900	67	0	TAC	-	-
*16S rRNA*	H	4901–6248	1348	0	-	-	-
*tRNA-Leu1*	H	6249–6312	64	0	TAG	-	-
*tRNA-Leu2*	H	6313–6381	69	0	TAA	-	-
*NAD1*	H	6382–7326	945	9	-	ATG	TAA
*tRNA-Pro*	H	7336–7402	67	−1	TGG	-	-
*NAD6*	H	7402–7896	495	10	-	ATG	TAA
*CytB*	H	7907–9046	1140	15	-	ATG	TAA
*tRNA-Ser2*	H	9062–9127	66	29	TGA	-	-
*tRNA-Thr*	L	9157–9227	71	10	TGT	-	-
*NAD4L*	H	9238–9534	297	−7	-	ATG	TAA
*NAD4*	H	9528–10,889	1362	10	-	ATG	TAA
*tRNA-His*	H	10,900–10,964	65	0	GTG	-	-
*NAD5*	H	10,965–12,692	1728	−21	-	ATG	TAA
*tRNA-Phe*	H	12,672–12,740	69	0	GAA	-	-
*CR*	-	12,741–13,264	524	0	-		
*COIII*	H	13,265–14,044	780	65	-	ATG	TAA
*tRNA-Lys*	H	14,110–14,176	68	10	TTT	-	-
*tRNA-Ala*	H	14,187–14,260	74	45	TGC	-	-
*tRNA-Arg*	H	14,306–14,374	69	10	TCG	-	-
*tRNA-Asn*	H	14,385–14,455	71	27	GTT	-	-
*tRNA-Ile*	H	14,483–14,552	70	5	GAT	-	-
*NAD3*	H	14,558–14,911	354	39	-	ATG	TAG
*tRNA-Ser1*	H	14,951–15,020	70	−1	GCT	-	-
*NAD2*	H	15,020–16,093	1074	3	-	ATG	TAG

**Table 3:** Nucleotide composition of the mitogenome of *P. reevei* and *P. aulanieri.*

Feature	Length	A%	T%	C%	G%	%A + T	%G + C	AT-Skew	GC-Skew
Whole mitogenome									
*P. reevei*	15,660	30.10	40.88	13.14	15.87	70.98	29.02	−0.152	0.094
*P. aulanieri*	16,096	29.48	39.37	14.83	16.32	68.85	31.15	−0.144	0.048
PCGs									
*P. reevei*	11,241	27.52	42.45	13.95	16.08	69.98	30.02	−0.213	0.071
*P. aulanieri*	11,256	26.87	40.70	15.67	16.76	67.57	32.43	−0.205	0.033
rRNAs									
*P. reevei*	2251	37.23	35.41	11.28	16.08	72.63	27.37	0.025	0.175
*P. aulanieri*	2308	36.74	34.84	11.92	16.51	71.58	28.42	0.027	0.162
tRNAs									
*P. reevei*	1465	35.77	36.04	11.95	16.25	71.81	28.19	−0.004	0.153
*P. aulanieri*	1485	34.28	35.35	12.93	17.44	69.63	30.37	−0.015	0.149
CR									
*P. reevei*	292	36.64	40.75	9.25	13.36	77.40	22.60	−0.053	0.182
*P. aulanieri*	524	33.40	38.17	16.60	11.83	71.56	28.44	−0.067	−0.168

**Table 4:** Codon count and relative synonymous codon usage in the mitochondrial genome of *P. reevei*. The asterisk (*) in the table indicates the stop codon.

Codon	Count	RSCU	Codon	Count	RSCU	Codon	Count	RSCU	Codon	Count	RSCU
UUU(F)	311	1.83	UCU(S)	120	2.59	UAU(Y)	108	1.59	UGU(C)	31	1.41
UUC(F)	29	0.17	UCC(S)	25	0.54	UAC(Y)	28	0.41	UGC(C)	13	0.59
UUA(L)	367	3.54	UCA(S)	56	1.21	UAA(*)	12	1.85	UGA(W)	75	1.44
UUG(L)	62	0.6	UCG(S)	9	0.19	UAG(*)	1	0.15	UGG(W)	29	0.56
CUU(L)	100	0.97	CCU(P)	74	2.24	CAU(H)	61	1.54	CGU(R)	22	1.54
CUC(L)	10	0.11	CCC(P)	17	0.52	CAC(H)	18	0.46	CGC(R)	6	0.42
CUA(L)	67	0.65	CCA(P)	31	0.94	CAA(Q)	69	1.84	CGA(R)	23	1.68
CUG(L)	14	0.14	CCG(P)	10	0.3	CAG(Q)	6	0.16	CGG(R)	5	0.35
AUU(I)	281	1.82	ACU(T)	83	2.17	AAU(N)	112	1.62	AGU(S)	60	1.3
AUC(I)	28	0.18	ACC(T)	16	0.39	AAC(N)	26	0.38	AGC(S)	19	0.41
AUA(M)	162	1.52	ACA(T)	47	1.23	AAA(K)	81	1.62	AGA(S)	62	1.34
AUG(M)	51	0.48	ACG(T)	8	0.21	AAG(K)	19	0.38	AGG(S)	19	0.41
GUU(V)	114	1.84	GCU(A)	146	2.51	GAU(D)	65	1.76	GGU(G)	81	1.39
GUC(V)	19	0.32	GCC(A)	19	0.35	GAC(D)	9	0.24	GGC(G)	23	0.4
GUA(V)	83	1.34	GCA(A)	60	1.04	GAA(E)	62	1.51	GGA(G)	70	1.22
GUG(V)	31	0.5	GCG(A)	6	0.1	GAG(E)	19	0.49	GGG(G)	57	0.99

**Table 5:** Comparison of mitogenome features in *Pomacea*, where (+) indicates presence and (−) indicates absence of the feature.

Feature	*P. reevei*	*P. aulanieri*	*P. diffusa*	*P. canaliculata*	*P. maculata*	*P. occulta*
Overlapping *NAD5*/*tRNA-Phe*	+	+	+	−	−	−
Overlapping *NAD1*/*tRNA-Pro*	−	−	−	+	+	+
ATP6 length (bp)	699	699	714	699	699	699
NAD1 length (bp)	939	945	945	960	960	960
NAD2 length (bp)	1074	1074	1071	1062	1062	1065
NAD4 length(bp)	1374	1362	1362	1368	1368	1368
NAD5 length (bp)	1707	1728	1728	1710	1710	1710
Start codon COIII	ATG	ATG	ATG	ATA	ATA	ATA
Range of tRNAs length (bp)	12	13	15	7	7	7
Tallo D en tRNA-Ser1	−	+	+	−	−	−
Tallo D en tRNA-Ser2	−	−	−	+	+	+
*12S rRNA* length (bp)	909	960	952	929	936	934
*16S rRNA* length (bp)	1340	1348	1346	1334	1336	1331

## Data Availability

All mitogenome sequences generated in this study were deposited in GenBank under accession numbers OR253802 and OR253803.

## Supplementary Materials

The following supporting information can be downloaded at https://www.mdpi.com/article/10.3390/genes14091769/s1, Figure S1: Shells of voucher specimens. A. *Pomacea reevei* Ampuero & Ramírez, 2023 (Voucher ID: AE152NBI, GenBank Accession Number: OR253802) from Bandeja Isla, Napo river, Loreto region, Peru (2°8′2.78″ S, 74°13′16.91″ W). B. *Pomacea aulanieri* (Deville & Hupé, 1850) (Voucher ID: Z23HYP, GenBank Accession Number: OR253803) from Paranapura river ca. Huallaga river, Yurimaguas, Loreto region, Peru (5°52′19.3″ S, 76°7′55.1″ W). Scale bars: 20 mm. Figure S2: Relative synonymous codon usages (RSCU) in the mitogenome of *P. aulanieri*. Figure S3: Secondary structure of the *12S rRNA* of *P. aulanieri*. Domains are indicated with Roman numbers and helices are numbered following Hickson et al. [27]. Figure S4: Secondary structure of the I–III domains of the *16S rRNA* of *P. aulanieri*. Domains are indicated with Roman numbers and helices are numbered following Wuys et al. [29]. Figure S5: Secondary structure of the IV–VI domains of the *16S rRNA* of *P. aulanieri*. Domains are indicated with Roman numbers and helices are numbered following Wuys et al. [29]. Figure S6: Secondary structures of the tRNA genes in the mitogenome of *P. aulanieri.* Figure S7: Evolutionary rate of the *Pomacea* mitogenomes published in GenBank and those from this work. Ka is the nonsynonymous nucleotide substitutions per nonsynonymous site, Ks is the synonymous nucleotide substitutions per synonymous site and Ka/Ks is ratio of nucleotide nonsynonymous to synonymous substitutions. Figure S8: Phylogenetic relationships of Caenogastropoda species based on protein-coding genes and ribosomal RNAs inferred using Maximum Likelihood (ML). Numbers on branches are bootstrap values. *Pomacea* species sequenced in this study are marked with an asterisk. Table S1: Codon count and relative synonymous codon usage in the mitochondrial genome of *P. aulanieri*. The asterisk (*) in the table indicates the stop codon.
