# Supplementary material for: Comparative Mitogenome Analysis of Two Native Apple Snail Species (Ampullariidae, Pomacea) from Peruvian Amazon
Source: Genes (Basel). 2023 Sep 7;14(9):1769. doi: 10.3390/genes14091769 (PMC10531094; doi:10.3390/genes14091769)
Supplement: Supplementary file 1 [file genes-14-01769-s001.zip › Table S1-02set23.pdf]

Supplementary Table S1. Codon count and relative synonymous codon usage in the mitochondrial genome of *P. aulanieri*. The asterisk (*) in the table indicates the stop codon.

Codon	Count	RSCU	Codon	Count	RSCU	Codon	Count	RSCU	Codon	Count	RSCU
UUU(F)	297	1.73	UCU(S)	120	2.54	UAU(Y)	109	1.48	UGU(C)	25	1.28
UUC(F)	47	0.27	UCC(S)	31	0.66	UAC(Y)	38	0.52	UGC(C)	14	0.72
UUA(L)	321	3.13	UCA(S)	48	1.02	UAA(*)	9	-	UGA(W)	68	1.3
UUG(L)	61	0.59	UCG(S)	7	0.13	UAG(*)	4	-	UGG(W)	36	0.7
CUU(L)	91	0.89	CCU(P)	89	2.68	CAU(H)	60	1.48	CGU(R)	23	1.71
CUC(L)	30	0.29	CCC(P)	10	0.33	CAC(H)	21	0.52	CGC(R)	1	0.07
CUA(L)	83	0.81	CCA(P)	23	0.72	CAA(Q)	61	1.56	CGA(R)	27	1.93
CUG(L)	30	0.29	CCG(P)	9	0.27	CAG(Q)	17	0.44	CGG(R)	4	0.29
AUU(I)	248	1.66	ACU(T)	73	1.77	AAU(N)	102	1.51	AGU(S)	38	0.81
AUC(I)	51	0.34	ACC(T)	25	0.61	AAC(N)	33	0.49	AGC(S)	36	0.77
AUA(M)	150	1.52	ACA(T)	55	1.33	AAA(K)	75	1.54	AGA(S)	68	1.45
AUG(M)	48	0.48	ACG(T)	12	0.29	AAG(K)	23	0.46	AGG(S)	29	0.62
GUU(V)	109	1.76	GCU(A)	130	2.21	GAU(D)	59	1.51	GGU(G)	72	1.25
GUC(V)	29	0.47	GCC(A)	34	0.58	GAC(D)	20	0.49	GGC(G)	25	0.43
GUA(V)	76	1.21	GCA(A)	59	1	GAA(E)	55	1.41	GGA(G)	70	1.21
GUG(V)	35	0.56	GCG(A)	12	0.2	GAG(E)	23	0.59	GGG(G)	64	1.11
