# Supplementary figures and images for: Comparative Mitogenome Analysis of Two Native Apple Snail Species (Ampullariidae, Pomacea) from Peruvian Amazon
Source: Genes (Basel). 2023 Sep 7;14(9):1769. doi: 10.3390/genes14091769 (PMC10531094; doi:10.3390/genes14091769)

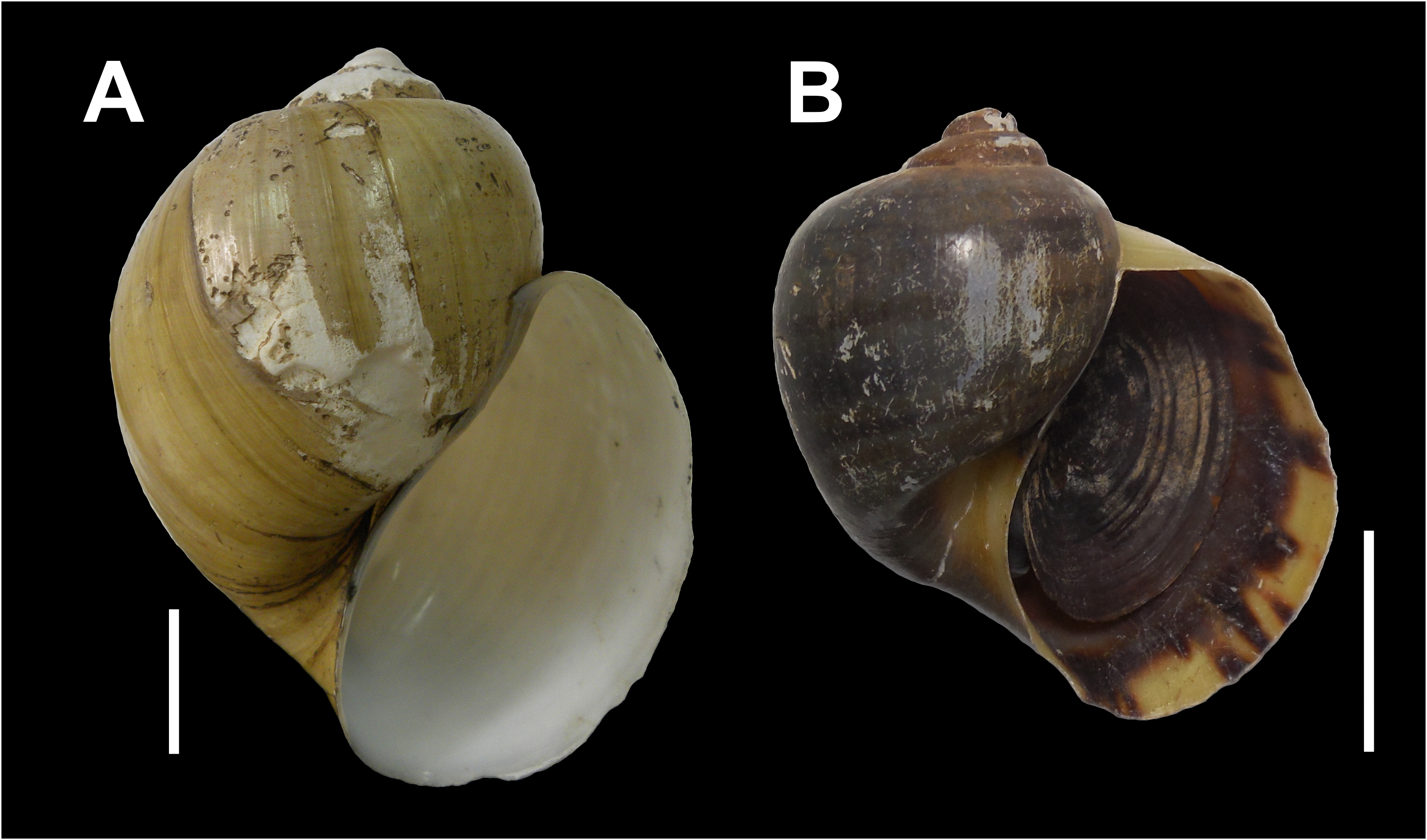

Supplement: Supplementary file 1 [file genes-14-01769-s001.zip › S1_Shells.jpg]

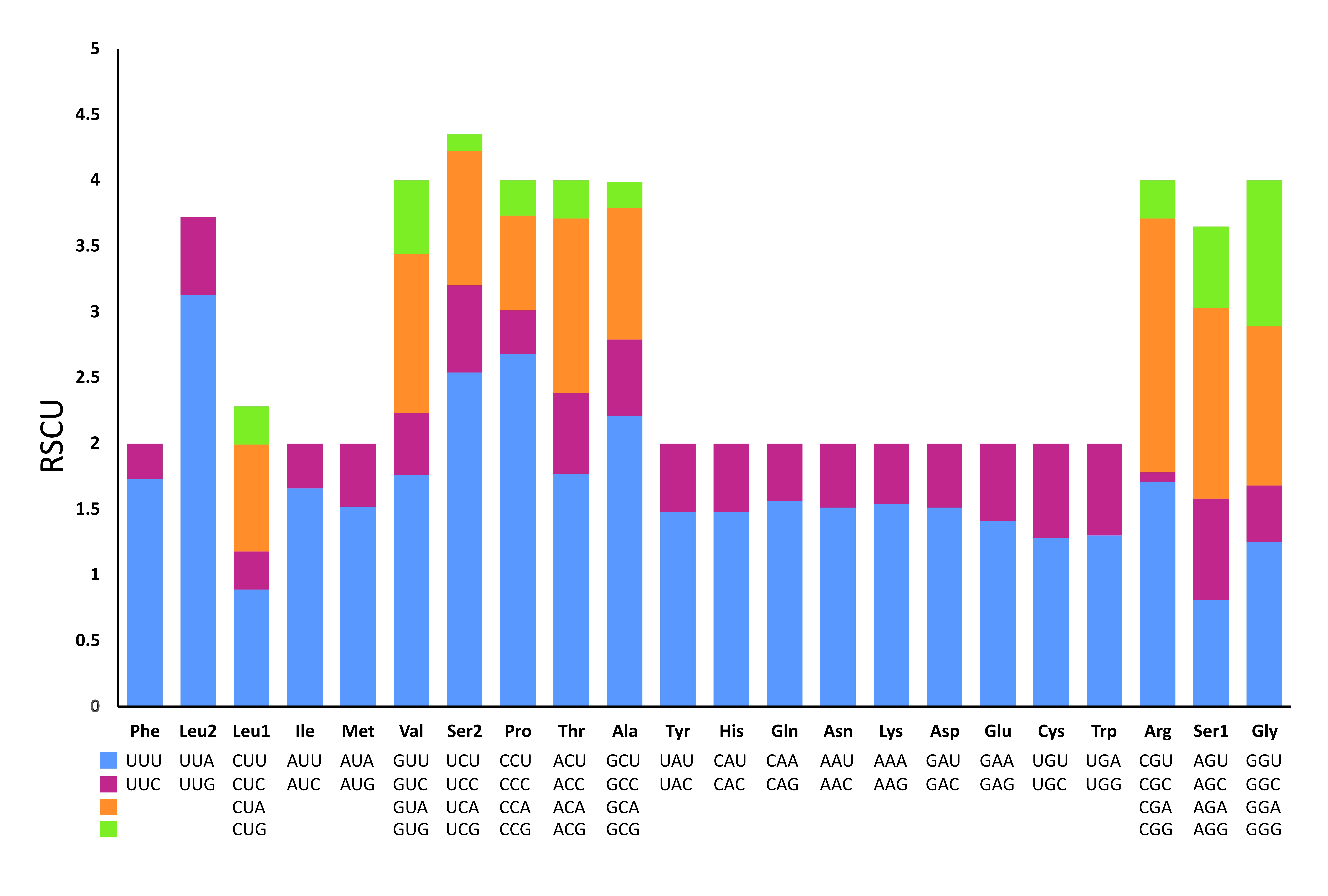

Supplement: Supplementary file 1 [file genes-14-01769-s001.zip › S2_RSCU_Paulanieri.jpg]

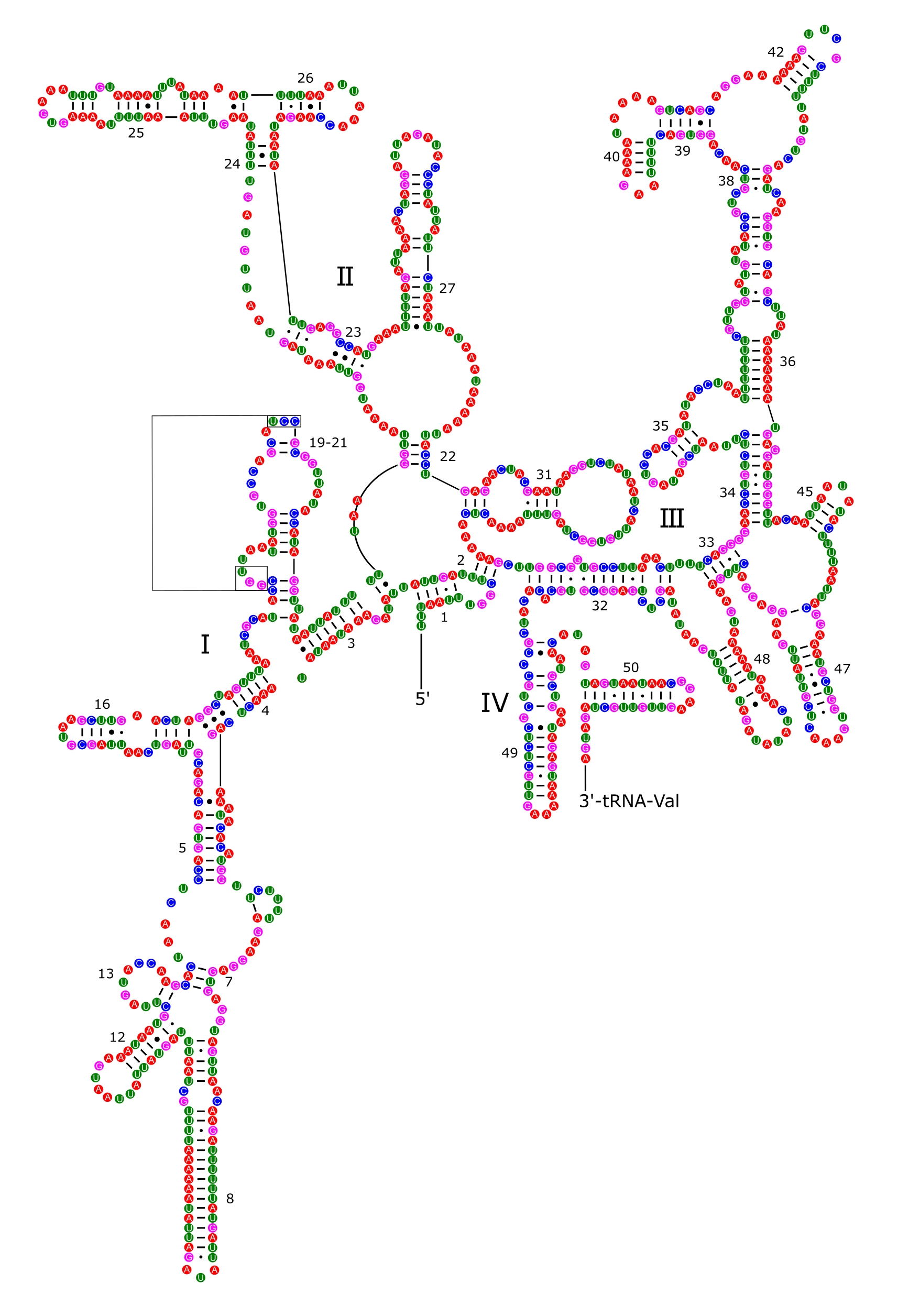

Supplement: Supplementary file 1 [file genes-14-01769-s001.zip › S3_12S_Paualanieri.jpg]

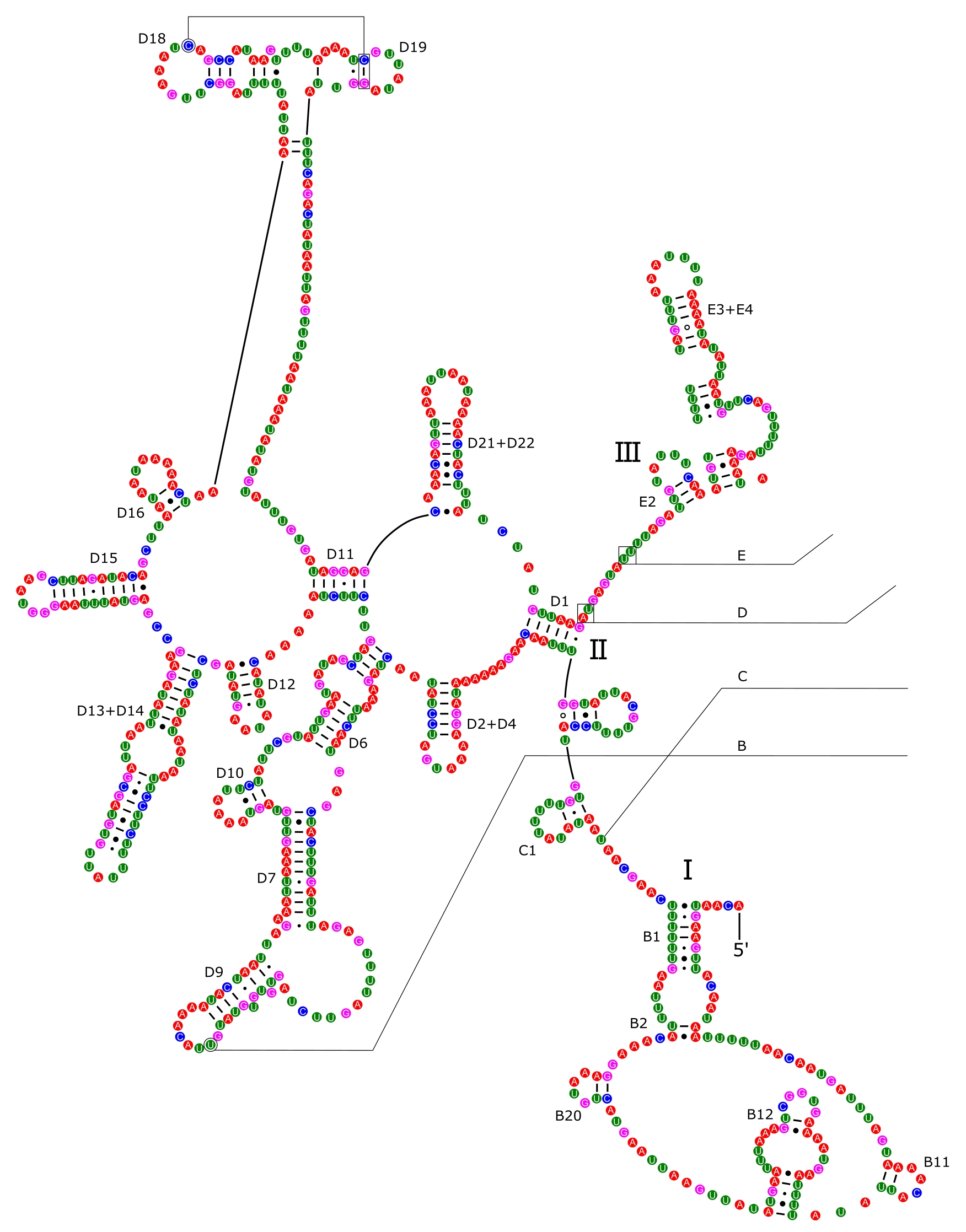

Supplement: Supplementary file 1 [file genes-14-01769-s001.zip › S4_16S_5prima_Paulanieri.jpg]

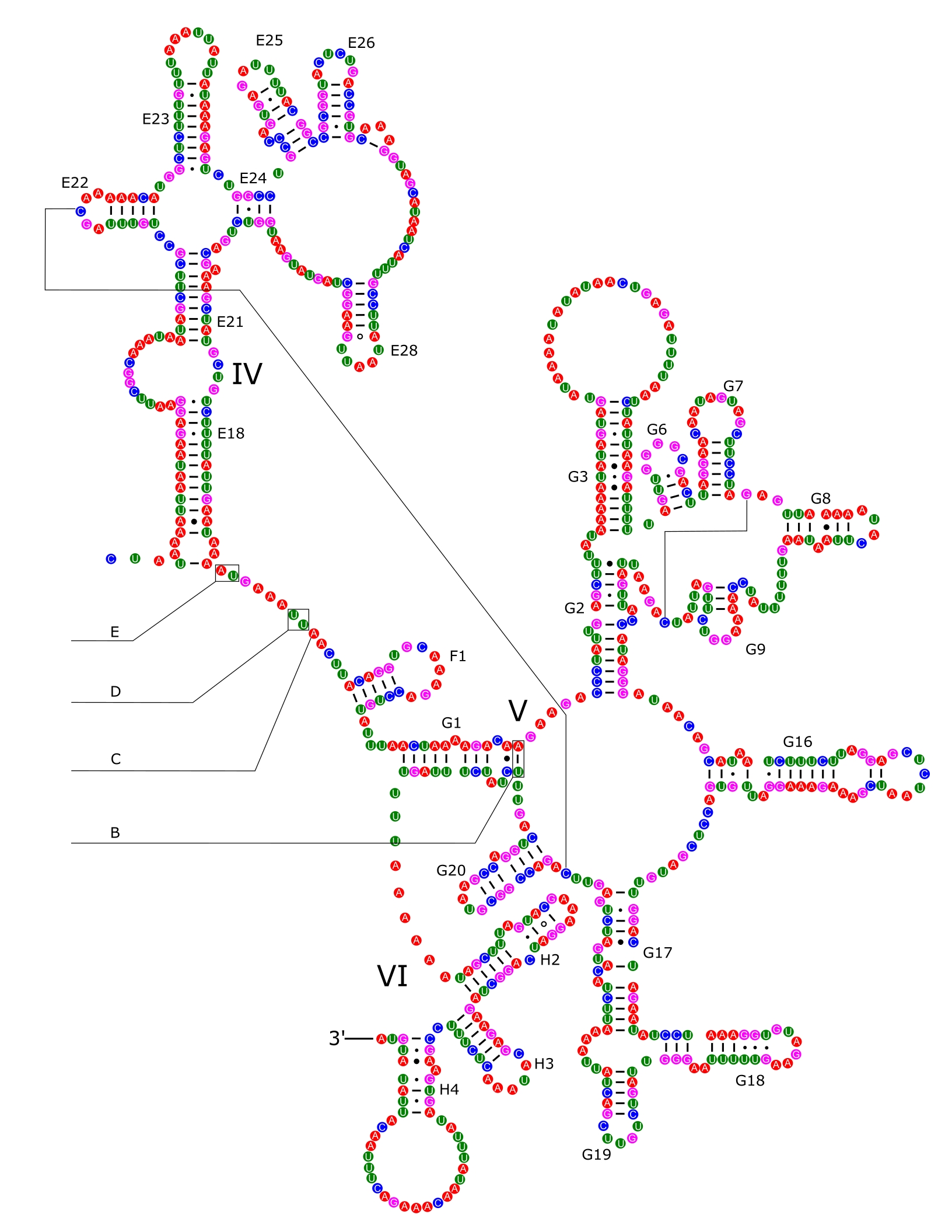

Supplement: Supplementary file 1 [file genes-14-01769-s001.zip › S5_16S_3prima_Paulanieri.jpg]

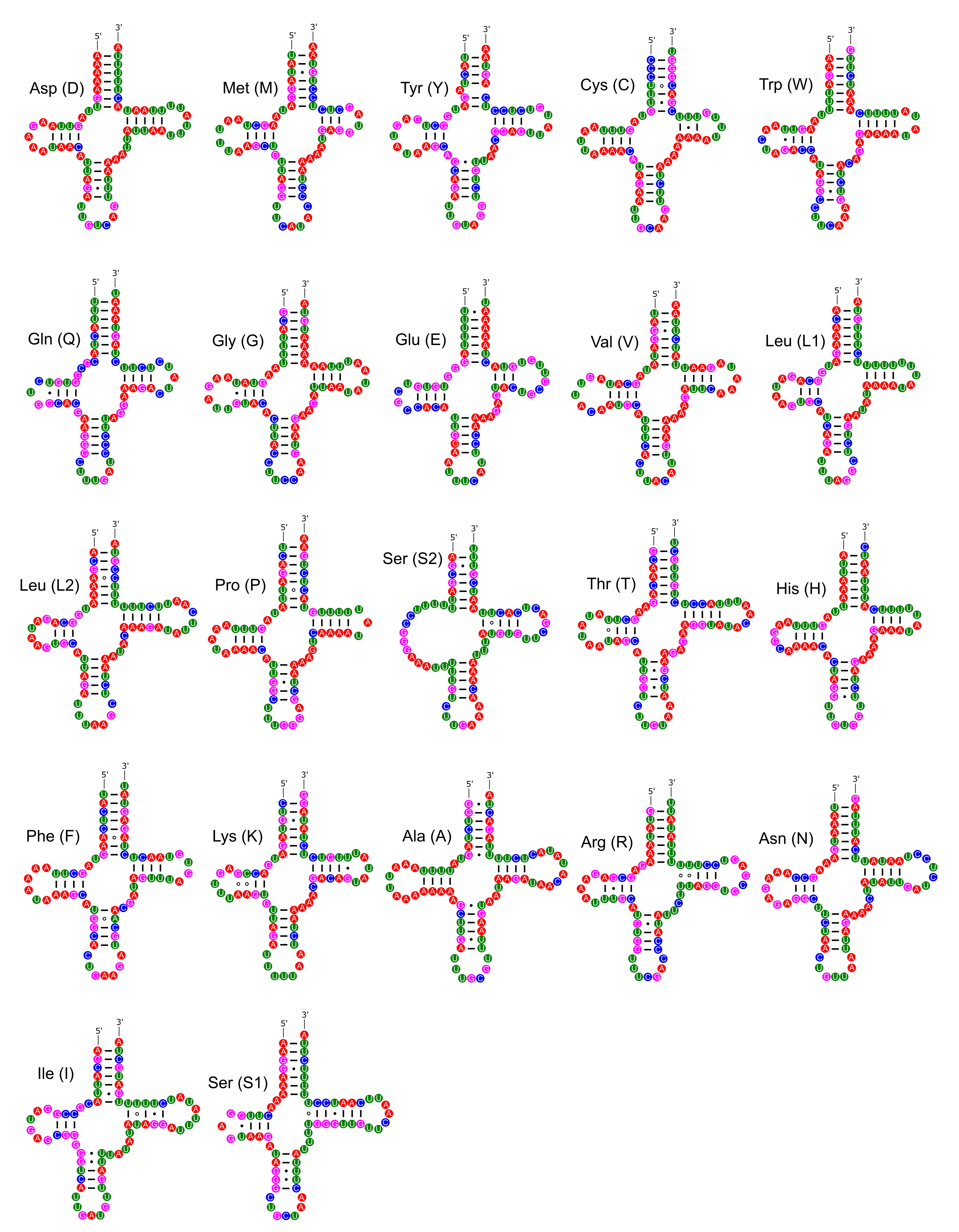

Supplement: Supplementary file 1 [file genes-14-01769-s001.zip › S6_ARNt_Paulanieri.jpg]

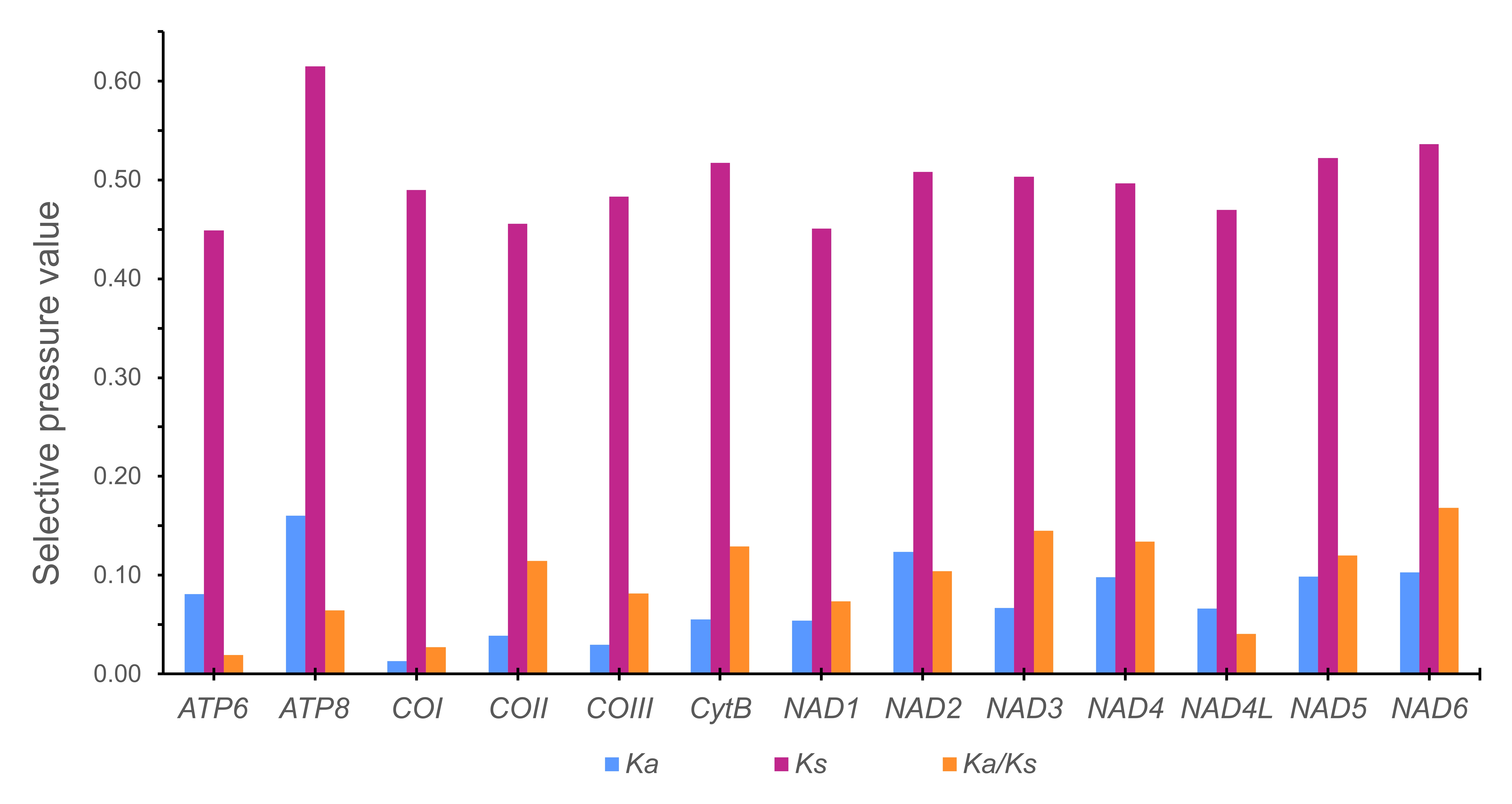

Supplement: Supplementary file 1 [file genes-14-01769-s001.zip › S7_KaKs.jpg]

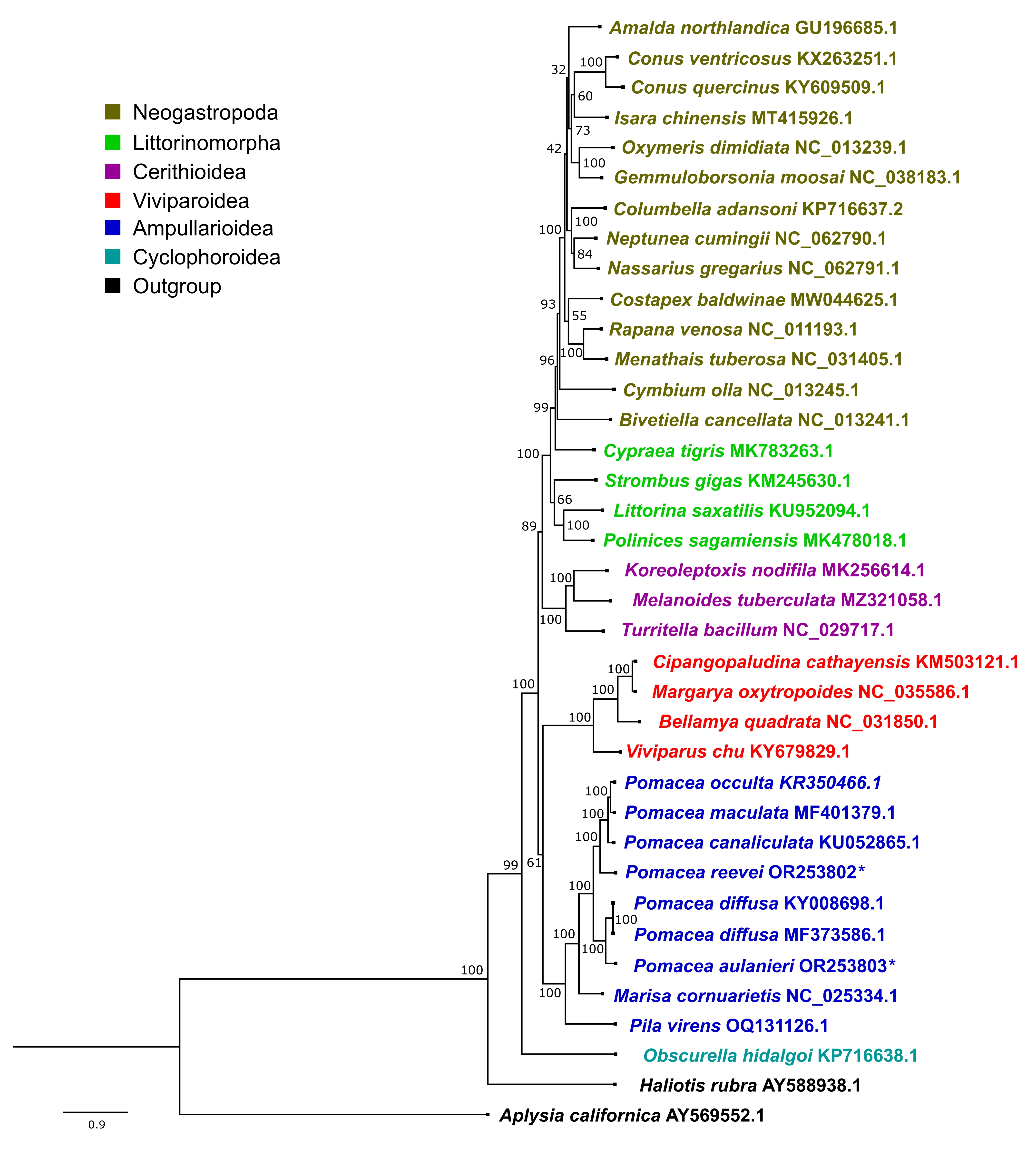

Supplement: Supplementary file 1 [file genes-14-01769-s001.zip › S8_Phylogeny_ML.jpg]
